# Polymerase η suppresses telomere defects induced by DNA damaging agents

**DOI:** 10.1093/nar/gku1030

**Published:** 2014-10-29

**Authors:** Hannah Pope-Varsalona, Fu-Jun Liu, Lynda Guzik, Patricia L. Opresko

**Affiliations:** 1 Department of Environmental and Occupational Health, University of Pittsburgh, Pittsburgh, PA 15219, USA; 2 McGowan Institute for Regenerative Medicine, University of Pittsburgh, Pittsburgh, PA 15260, USA; 3 Center for Nucleic Acids Science and Technology, Carnegie Mellon University, Pittsburgh, PA 15213, USA

## Abstract

Telomeres at chromosome ends are normally masked from proteins that signal and repair DNA double strand breaks (DSBs). Bulky DNA lesions can cause DSBs if they block DNA replication, unless they are bypassed by translesion (TLS) DNA polymerases. Here, we investigated roles for TLS polymerase η, (polη) in preserving telomeres following acute physical UVC exposure and chronic chemical Cr(VI) exposure, which both induce blocking lesions. We report that polη protects against cytotoxicity and replication stress caused by Cr(VI), similar to results with ultraviolet C light (UVC). Both exposures induce ataxia telangiectasia and Rad3-related (ATR) kinase and polη accumulation into nuclear foci and localization to individual telomeres, consistent with replication fork stalling at DNA lesions. Polη-deficient cells exhibited greater numbers of telomeres that co-localized with DSB response proteins after exposures. Furthermore, the genotoxic exposures induced telomere aberrations associated with failures in telomere replication that were suppressed by polη. We propose that polη's ability to bypass bulky DNA lesions at telomeres is critical for proper telomere replication following genotoxic exposures.

## INTRODUCTION

Human telomeres are 5–15 kb of TTAGGG/CCCTAA tandem repeats at chromosome ends. The protein complex that binds telomeres, shelterin, functions with telomere structure to provide a protective cap to chromosome ends (reviewed in (1)). Dysfunctional telomeres are recognized as a DNA double strand break (DSB), thereby signaling the recruitment of DNA damage signaling and repair proteins to the chromosome end (2). Increasing evidence indicates that telomeres are hypersensitive to DNA replication stress induced either by polymerase inhibition with aphidicolin, oncogene expression or deficiencies in proteins that stabilize stalled replication forks including ATR kinase and specialized DNA helicases (3–7). These studies reveal that replication stress in cells leads to telomere aberrations that manifest on metaphase chromosomes as multitelomeric signals at a chromatid end (doublet) or a telomere signal-free end (telomere loss). Replication stress is commonly defined as the slowing or stalling of replication fork progression due to obstacles or decreased DNA synthesis, and can activate a stress response in the cell (8). Evidence indicates that stalled replication forks can collapse into DNA DSBs (8), which may be particularly detrimental at telomeres given that DSB repair pathways are normally suppressed by telomeric shelterin (9–11). Recent findings indicate that as few as five dysfunctional telomeres are enough to provoke cellular senescence (12), demonstrating the importance of maintaining telomere integrity.

Replication stress can also be induced at specific loci within the genome if the replication fork encounters a DNA lesion. Bulky lesions left unrepaired can block the replication machinery and signal the recruitment of translesion (TLS) DNA polymerases. The TLS polymerase extends DNA synthesis across the lesion, and prevents replication fork demise, allowing the cell to complete genome replication so the lesion can be repaired at a later time (reviewed in (13)). TLS is a DNA damage tolerance mechanism with the caveat that it may not be error free, and may introduce mutations. DNA polymerase η (polη) is distinguished for its efficiency in inserting correct nucleotides opposite UV-induced *cis-syn* cyclobutane pyrimidine dimers (CPD), the most frequent UV photoproducts (14–16). Mutations in the *POLH* gene, which encodes polη, cause a rare autosomal recessive disorder called xeroderma pigmentosum group variant (XPV), characterized by sunlight sensitivity and a high incidence of UV-induced skin cancers (14). Cells from XPV donors have normal nucleotide excision repair (NER) and can remove UV photoproducts, but exhibit increased UV-induced replication stress (17,18), mutagenesis (19) and chromatid breaks (20). Homologous recombination (HR) serves as an alternative mechanism for bypassing DNA lesions or for repairing collapsed replication forks at blocking lesions (21). However, numerous studies indicate that TRF2 and other shelterin factors repress HR repair proteins, protecting telomeres from aberrant processing or lengthening by the ALT pathway (reviewed in (1)). Additionally, polη is required for successful replication at common fragile sites (22). Telomeres resemble common fragile sites in that they are difficult to replicate and sensitive to aphidicolin (3). However, roles for TLS polymerases in telomere preservation remain unexamined.

Previous studies show that telomeres are susceptible to genotoxic exposures that induce bulky lesions. Ultraviolet light causes bulky CPDs, which are either repaired by NER or bypassed by DNA polη if the lesion stalls replication at the fork. Telomere sequences contain hot spots for UV pyrimidine dimers on both the G-rich and C-rich strands (23,24). A recent study reported evidence that telomeres are deficient in CPD removal (24). While UVB exposures of human cells did not alter mean telomere lengths (24), the impact of UV on individual telomeres is unknown. Hexavalent chromium (Cr(VI)) is another environmental genotoxic agent that induces a spectrum of adducts including bulky lesions that are repaired by NER (25). Evidence indicates that Cr(VI) preferentially reacts with guanine runs (26), which predicts that telomeres are also susceptible to Cr(VI)-induced lesions. Consistent with this, we previously reported that Cr(VI)-induced replication stress causes telomere loss and aberrations (27). Furthermore, Cr(VI) exposure in *Saccharomyces cerevisiae* indicate that polη protects against Cr(VI)-induced mutagenesis (28).

In this study, we investigated a role for polη in telomere preservation following an acute physical (UVC) or chronic chemical (Cr(VI)) exposure that generates bulky DNA lesions in telomeric sequences. We demonstrate that replication stress is induced at the telomeres following these exposures, which also triggered the accumulation of polη at telomeric regions. Furthermore, we demonstrate that these genotoxic exposures in cells lacking functional polη cause increased telomere aberrations associated with failures in telomere replication. Thus, we uncovered evidence that a TLS DNA polymerase is necessary to defend telomeres against the effects of bulky DNA lesions.

## MATERIALS AND METHODS

### Cell culture and exposures

SV40-transformed XP30RO human fibroblasts carrying an empty vector (pCDNA) or complemented with pCDNA-polη were a generous gift from Alan Lehmann, University of Sussex. The XP30RO cells have a homozygous deletion near to the 5′ end of the *POLH* gene which causes extensive truncation of the polη protein (14). U2OS cell lines expressing an enhanced green fluorescent protein (eGFP)-polη construct were obtained by Fugene^®^ HD Transfection Reagent according to the manufacturer's instructions. U2OS cells stably expressing GFP-ATR were a generous gift from Jiri Lukas. GFP-polη XPV cells were a gift from Alan Lehmann (29). BJ primary skin fibroblasts derived from a normal individual were from ATCC. XPV (GM02359) primary skin fibroblasts derived from an individual homozygous for a C to T transition at nucleotide 1117 of the POLH gene resulting in a premature stop codon was from the Coriell Cell Repository. Cells were cultured in Dulbecco's modified Eagle medium supplemented with 10% fetal bovine serum and penicillin (50 units/ml), and streptomycin (50 units/ml) in humidified chambers with 5% CO_2_ and 20% O_2_ at 37°C.

Cells were exposed to K_2_Cr_2_O_7_ (Sigma-Aldrich, St. Louis, MO, USA) as described previously (27), for 48 h at indicated concentrations. Cells were irradiated with 254 nm UVC light at 0, 5 and 10 J/m^2^ UVC with a fluence of 1 J/m^2^/s as measured with a UVX31 meter. Recovery was conducted in fresh Cr(VI)-free media at 37°C for specified incubation times.

### Cell survival assay

Cellular toxicity was determined by a cell counting assay using the Beckman Coulter™ Z1 Coulter^®^ Particle Counter (aperture 100 μm). Cells were seeded at a density of 1 × 10^5^ cells per dish in 35-mm culture dishes and incubated for 24 h. Cells were then exposed to either Cr(VI) for 48 h at various concentrations or to UVC at various doses as indicated and were allowed to recover for 6 h. Cells were then counted and subcultured at 4 × 10^4^ cells per 10-cm culture dish. Following a 7-day subculture in Cr(VI)-free media, cells were recounted.

### Flow cytometry

Cell cycle profiles were obtained using Click-iT® EdU Flow Cytometry Cell Proliferation Assay (Life Technologies™) according to manufacturer's instructions. Briefly, 2.5 × 10^5^ cells were seeded in 10-cm culture dishes 24 h prior to exposures. Cells were exposed to either UVC or Cr(VI) as described and incubated with 10 μM Click-iT® EdU 1 h prior to harvest for each time point. Cells were harvested, counted and then resuspended in 1% bovine serum albumin (100 μl/1 × 10^6^ cells). Next, cells were fixed and stored at 4°C overnight in an ice slurry. After cells were permeabilized and incubated with the reaction cocktail, they were stained with DAPI for DNA content. Detection of Click-iT® EdU performed by flow cytometry with BD FACSAria II.

### Immunofluorescene-fluorescence *in situ* hybridization (IF-FISH)

As previously described (27), IF-FISH was performed either immediately after Cr(VI) exposure or after 6 h recovery from UVC exposure. Cells were fixed in 2% paraformaldehyde for 15 min followed by permeabilization in 0.2% Triton X-100 for 10 min. Cells were then blocked in 1 mg/ml bovine serum albumin, 3% fetal bovine serum, 0.1% Triton X-100, 1 mM ethylenediaminetetraacetic acid (pH 8.0) in phosphate buffered saline for 1 h and immuno-stained with rabbit anti-GFP polyclonal antibody (1:400; GeneTex, Irvine, CA, USA), anti-phospho-Histone H2AX (1:500, Millipore) or anti-53BP1 (1:500, Cell Signaling Technology). Next, cells were incubated with either Alexa 488-conjugated (Invitrogen, 1:500) goat anti-mouse secondary antibody or Cy5-conjugated goat anti-mouse (JIR laboratories, Inc., 1:400). Cells were fixed in 2% paraformaldehyde for 5 min and dehydrated in 70, 95, 100% ethanol for 5 min each. Samples were denatured for 10 min at 80°C in hybridization solution (70% deionized formamide, 10% NEN blocking reagent [Roche], 0.1 M Tris-HCl [pH 7.4], MgCl_2_ buffer [82 mM NaH_2_PO_4_, 9 mM citric acid, 20 mM MgCl_2_] and 0.5 mg/ml Cy3-OO-(CCCTAA)_3_ PNA probe [Panagene, South Korea]). Samples were hybridized for 2 h at room temperature and washed twice in 70% deionized formamide and 10 mM Tris-HCl [pH 7.4]. Samples were counterstained with DAPI and images were acquired with a Nikon A1 confocal microscope.

### Chromosomal telomere fluorescent in situ hybridization (Telomere FISH)

Cells were seeded (3 × 10^5^ for Cr(VI)-treated or 8 × 10^5^ for UVC-treated) in 10-cm culture dishes 24 h before exposure. After exposures, cells were treated with 0.05 μg/ml colcemid (Invitrogen) for 8 h. As previously described (27), Telomere FISH was executed on metaphase spreads. Cells were harvested and incubated with 75 mM KCl hypotonic buffer for 12 min at 37°C. Cells were then fixed and stored in 3:1 methanol/acetic acid. Cells were dropped onto slides and set overnight. Cells were then fixed in 4% formaldehyde for 2 min, washed in PBS and incubated with 0.1% pepsin in 0.01 N HCl for 10 min at 37°C. Cells were fixed, washed and then dehydrated in 70, 90 and 100% of ethanol for 5 min. Samples were by air-dried and then denatured at 80°C for 3 min in hybridization solution (see IF-FISH). Samples were hybridized for 2 h at room temperature, washed twice for 20 min each with wash solution I (70% deionized formamide, 10 mM Tris-HCl [pH 7.4] and 0.01% bovine serum albumin) and three times 15 min each with wash solution II (100 mM Tris-HCl [pH 7.4], 66.7 mM NaCl and 0.1% Tween 20). Finally, slides were stained with DAPI and mounted with coverslips.A Nikon Ti90 epifluorescence microscope (Nikon Inc., NY, USA) equipped with PlanApo 606/1.40 oil immersion objective was used to image metaphase chromosomes. Images were obtained and analyzed with NIS element advanced software using the same settings for set of cell lines in each experiment. A series of z-stacked images (0.15-mm steps) were acquired for the identification and examination of telomere signal-free chromosome ends, doublets and aberrations for each metaphase.

### Statistical methods

OriginPro 8 software was employed for all statistical analyses. Two-sample *t*-test for variance was used to determine significance of mean differences between two treatments or time points. One-way ANOVA followed by the Holm–Sidak test for means comparison test determined significance of differences among more than two treatments or time points. The statistically significant level was set at *P* < 0.05.

## RESULTS

### Polymerase η deficiency causes increased sensitivity to UVC and Cr(VI) exposures

**Figure 1.**
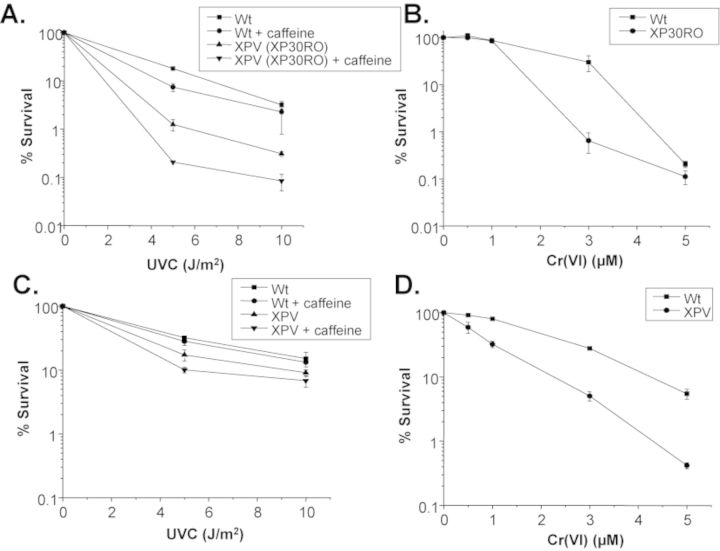
Analysis of the sensitivity of polη-deficient cells to UV and Cr(VI). After indicated UVC irradiation and 6 h recovery, or Cr(VI) exposure for 48 h, cells were subcultured in medium (without Cr(VI)) for 8 days and then counted using a Coulter counter. (**A**) UVC sensitivity of SV40 immortalized XP30RO-derived cells with vector alone or expressing polη (Wt) in the presence or absence of 0.38 mM caffeine. (**B**) Cr(VI) exposure sensitivity of SV40 immortalized XP30RO-derived cells with vector or expressing polη (Wt). (**C**) UVC sensitivity of XPV (GM02359) and BJ primary fibroblasts exposed with or without 0.38 mM caffeine. (**D**) Cr(VI) exposure sensitivity of XPV (GM02359) and BJ cells. Percent survival was determined by dividing the number of cells at each exposure by the number of cells in the untreated sample. Values represent the mean ± SE from two to five independent experiments for each survival assay.

To test for a potential role for polη in preserving telomeres after genotoxic stress we chose to examine previously established and well-characterized isogenic cell lines that are proficient or deficient for polη. SV40-transformed XP30RO human fibroblasts complemented with a polη expression vector (Wt) or vector alone (XPV) were generously provided by Dr Alan Lehmann (University of Sussex). We first confirmed that XPV cells show increased sensitivity to UVC (30) (Figure 1A). Following UVC exposures and 6 h of recovery, the cells were sub-cultured and allowed to recover for 8 days in fresh media, and then counted. Caffeine addition enhanced UVC sensitivity of XPV cells but not Wt cells, as previously shown (31). Polymerase η (polη) deficient cells are also hypersensitive to DNA replication stress induced by hydroxyurea and chemotherapeutic agents, including cisplatin and gemcitabine (30,32,33). We and others showed that Cr(VI) exposure also causes replication stress and replication-dependent chromosome breaks (27,34–36). Therefore, we predicted that polη might similarly protect against Cr(VI)-induced cytotoxicity. Cells were exposed to various concentrations of Cr(VI) for 48 h, followed by recovery for 8 days in Cr(VI)-free media. At 3 μM Cr(VI) exposure XPV cells exhibited a dramatic increase in sensitivity, compared to Wt cells, as indicated by a 42-fold decrease in relative cell number (Figure 1B). Similar results were obtained in primary cell lines from XPV patients (GM02359) compared to normal human fibroblasts (BJ) (Figure 1C and D). We observed a 5.5-fold decrease in XPV cells after 3 μM Cr(VI) and a 13-fold decrease after 5 μM Cr(VI), compared to normal BJ cells. In general, the SV40-transformed cells exhibited greater sensitivity to UVC and Cr(VI) compared to the primary cells, likely due to SV40 large T antigen suppression of p53 protein, as described previously for UVC (20,37). In conclusion, our results identify a novel role for polη in suppressing cytotoxicity following Cr(VI) exposure and suggest that polη TLS protects against Cr(VI)-induced replication stress, similar to its role following UVC exposures.

### Polη-deficient cells show delayed recovery from genotoxic-induced inhibition of DNA replication

**Figure 2.**
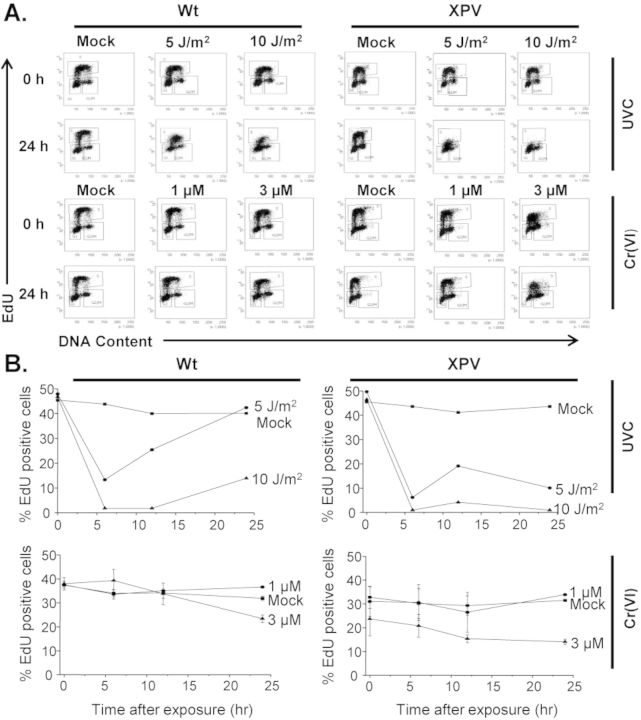
Cell cycle profiles of Wt and XPV cells after UVC exposure or Cr(VI) exposure. Cell cycle profiles of SV40 immortalized XP30RO-derived cells expressing polη (Wt) or vector alone (XPV). Cells were irradiated with 0, 5 or 10 J/m^2^ UVC or exposed to 0, 1 or 3 μM Cr(VI) for 48 h and then allowed to recover in fresh media. Cells were labeled with EdU 1 h prior to harvesting at the various recovery time points and analyzed by flow cytometry. (**A**) Dot plots of G_1_, S, G_2_/M phases of the cell cycle show DNA content on the *x* axis and EdU incorporation on the *y* axis. (**B**) Quantitative analysis of percent of cells actively incorporating EdU at the indicated recovery time points. Values for Cr(VI) represent the mean ± SE from two independent experiments.

Polη-deficient cells are known to exhibit a longer UV-induced S-phase delay compared to normal cells, due to polη's essential role in resumption of DNA replication following UV exposure (20,30). Next, we examined cell cycle profiles to confirm that polη complementation of XP30RO protects against UV-induced replication stress, and to test whether polη also suppresses Cr(VI)-induced replication stress. We expected XPV cells would show a reduced fraction of DNA replicating cells compared to Wt cells following recovery from UVC and Cr(VI) exposures. We obtained cell cycle profiles by fluorescence-activated cell sorting (FACS) analysis of DNA content and identified cells undergoing DNA replication by EdU pulse labeling prior to harvesting at each recovery time point. To ensure data collection was from live cells, we simultaneously stained cells with LIVE/DEAD^®^ Fixable dyes to eliminate any dead cells (Supplementary Figure S1). After 5 and 10 J/m^2^ UVC exposures, both polη-proficient and -deficient cells show a reduction in the fraction of EdU-positive cells at 6-h recovery (Figure 2). By 24-h recovery, Wt cells exposed to 5 J/m^2^ showed complete recovery of EdU-positive cells to pre-exposure levels, while those exposed to 10 J/m^2^ had increased but not yet fully recovered. In contrast, for XPV cells, both UVC exposures induced a greater reduction in the fraction of EdU-positive cells, compared to Wt cells, at 12- and 24-h recovery. Our results confirm that polη is essential for normal recovery of DNA replication and cell cycle progression after UVC exposure, consistent with previous studies (30) (Figure 2).

Cr(VI) was shown to inhibit DNA replication and cause cell cycle arrest during exposure (36,38). To test if polη has a role in the recovery from Cr(VI)-induced replication stress, we examined the fraction of cells replicating DNA at various time points following 48 h of low Cr(VI) levels. Wt cells exposed to 1 μM Cr(VI) exhibited a similar fraction of EdU-positive cells compared to untreated cells. However, following 3 μM Cr(VI) exposure, these cells show a reduction in EdU-positive cells by 12-h recovery progressing to greater reduction by 24 h. XPV cells exposed to 1 μM Cr(VI) showed a slight reduction in EdU-positive cells at 12 h post exposure, but recovered to pre-exposure levels by 24-h recovery. XPV cells exposed to 3 μM Cr(VI) showed fewer EdU-positive cells at 0-h recovery compared to untreated cells, and did not recover by 24 h post exposure. In summary, we observed a greater reduction in cells replicating DNA following low Cr(VI) exposures in the absence of polη. This suggests that TLS synthesis, as with UV lesions, is important in replication recovery from Cr(VI)-induced DNA lesions.

### UVC and Cr(VI) exposures induce ATR localization to telomeres

**Figure 3.**
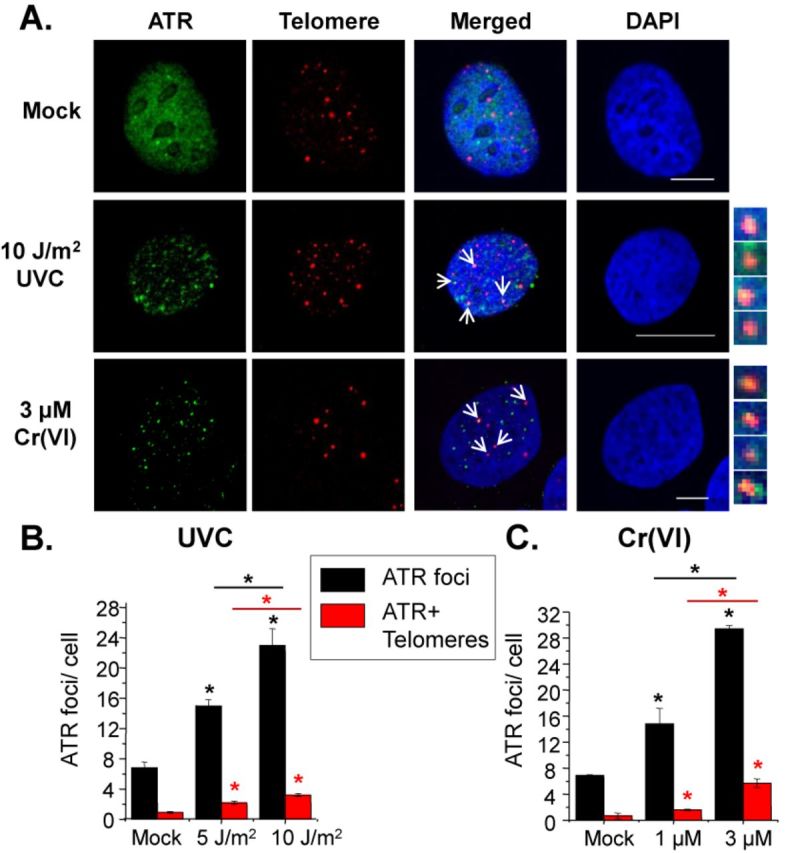
UVC and Cr(VI) induce replication stress at telomeres. (**A**) Confocal images of eGFP-ATR U2OS cells exposed to UVC and allowed to recover for 6 h or exposed to Cr(VI) for 48 h. Cells were analyzed via IF-FISH of ATR (green) and telomere (red) co-localization (yellow). Average ATR foci and co-localized ATR and telomere foci per cell after indicated UVC dose (**B**) or Cr(VI) concentration (**C**). The data represent mean ± SE from two experiments and approximately 50 interphase cells. Significant difference from the mock is indicated with * above the bar, and significant difference between exposures is indicated with a * above the line; black lines refer to ATR foci comparisons, red lines refer to ATR+ telomere foci (*P* < 0.05). Bars, 10 μM.

Having confirmed that UVC and Cr(VI) impact DNA replication, we next asked whether these exposures cause replication stress at telomeres. Cell cycle checkpoint activation leads to inhibition of cell cycle progression (reviewed in (39)). Ataxia telangiectasia and Rad3-related kinase protein (ATR) activation represents one of the initial signals for S-phase checkpoint activation. ATR is activated by RPA-bound single-stranded DNA at sites of polymerase stalling (40). Previous reports indicate that ATR is activated following UVC or Cr(VI) exposures (38,41), and that ATR is required for telomere maintenance (5,42). Since ATR localization to stressed replication forks is well established (43), we reasoned that ATR co-localization with telomeric DNA would serve as an indicator of replication stress at telomeres. For this we used the IF-FISH assay to stain telomeric DNA in U2OS cells that stably express eGFP-ATR (provided by Dr Jiri Lukas, University of Copenhagen). Cells irradiated with UVC were allowed to recover for 6 h before processing and imaging by confocal microscopy. UVC exposures induced a dose-dependent increase in ATR foci formation (Figure 3). The average ATR foci per cell increased 2- and 3-fold after 5 and 10 J/m^2^ UVC, respectively, compared to mock exposure (Figure 3B). An average of two to three ATR foci co-localized with telomeric DNA after UVC.

We then examined whether ATR formed foci after Cr(VI) treatment. Similar to results with UVC, cells treated with Cr(VI) for 48 h showed concentration-dependent increases in the amount of ATR foci (Figure 3C). We observed a 2-fold or greater than 4-fold increase in ATR foci per cell after 1 or 3 μM Cr(VI), respectively, compared to mock exposures. On average, one or two ATR foci localized to telomeres after 1 μM Cr(VI), while greater than four ATR foci co-localized to telomeres after 3 μM Cr(VI). Taken together, these results provide evidence that both UVC and Cr(VI) exposures induce replication stress at telomeric regions.

### UVC and Cr(VI) induce polη foci formation and localization to telomeres

**Figure 4.**
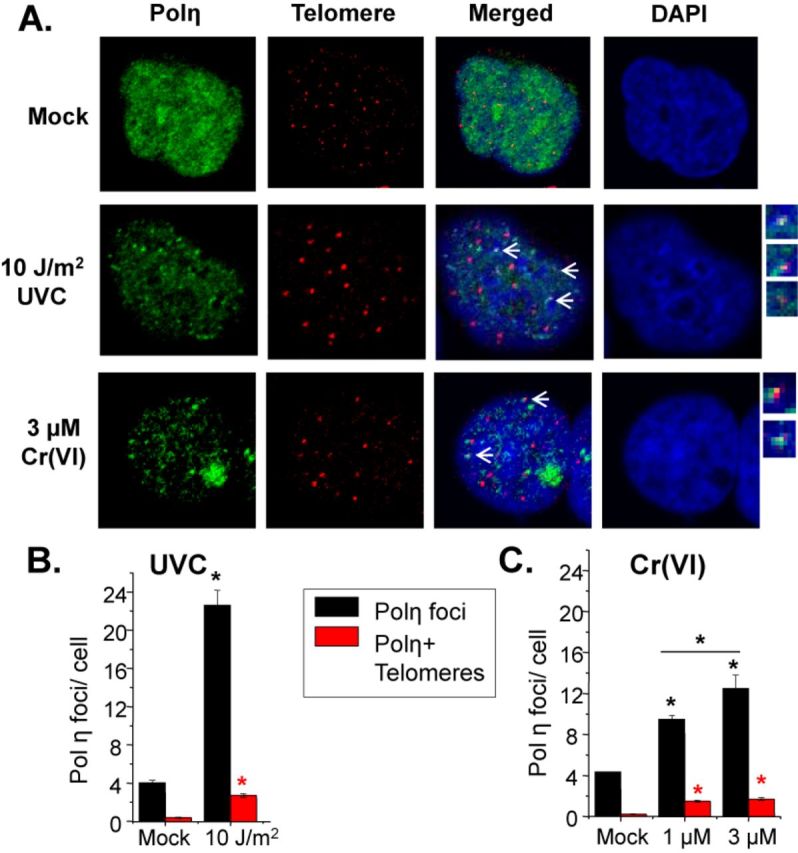
UVC and Cr(VI) induce polη localization to telomeres. (**A**) Confocal images of eGFP-Polη XP30RO cells exposed to UVC and allowed to recover for 6 h or exposed to Cr(VI) for 48 h. Cells were analyzed via IF-FISH of polη (green) and telomere (red) co-localization (yellow). Average polη foci and co-localized polη and telomere foci per cell after indicated UVC dose (**B**) or Cr(VI) concentration (**C**). The data represent mean ± SE from two independent experiments and a minimum of 50 interphase cells. Bars with a symbol of * indicates a significant difference compared to mock exposure and between the different exposures (*P* < 0.05).

Polη accumulates in nuclear foci after UVC irradiation at sites of unrepaired DNA lesions and stalled replication forks (44). To study the localization of polη to telomeres after UVC or Cr(VI) treatment, we used SV40-transformed XP30RO cells that stably express eGFP-Polη (a gift from Dr Alan Lehmann, University of Sussex (44) and IF-FISH (Figure 4A). Cells were exposed to 0 (mock) or 10 J/m^2^ UVC and incubated for 6 h before being processed for IF-FISH. In agreement with previous studies, we confirmed that UVC increases polη foci formation, and observed a 5-fold increase in polη foci per cell compared to mock treatment (Figure 4B). After 10 J/m^2^ UVC, an average of two polη foci co-localized to telomeric regions per cell. We obtained similar results for UVC-induced polη localization to telomeres in telomerase-negative human U2OS cells (Supplementary Figure S2). This represents the first report of polη localization to telomeres.

Cr(VI) exposures for 48 h also induced a concentration-dependent increase in polη foci formation (Figure 4C). We observed a 2-fold or 3-fold increase in polη foci per cell following 1 or 3 μM Cr(VI), respectively, compared to mock treatment. Cr(VI) exposures induced between one to two co-localized polη and telomere foci per cell. These results indicate that in addition to polη's established role in responding to UVC, polη responds to DNA lesions induced by low level Cr(VI) exposure. Moreover, these results demonstrate polη's ability to access telomeric DNA after both physical and chemical genotoxic exposures, and suggest that polη may respond to stalled replication forks at telomeres.

### Polη suppresses DNA damage signaling at telomeres

**Figure 5.**
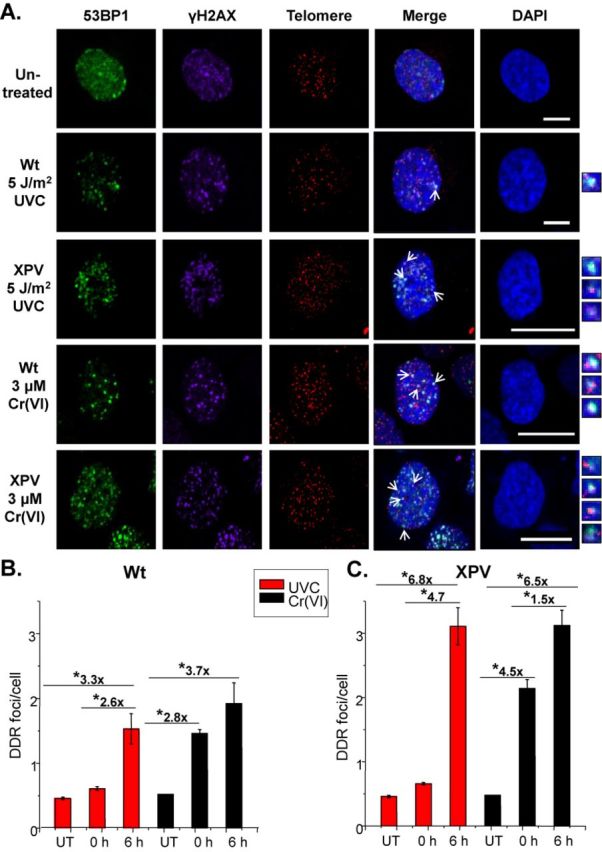
UVC and Cr(VI) induce a DDR at telomeres. SV40 immortalized XP30RO-derived cells expressing polη (Wt) or vector alone (XPV) following irradiation with 5 J/m^2^ UVC or 3 μM Cr(VI) for 48 h. Cells were analyzed via IF-FISH for 53BP1 (green), γH2AX (magenta) and telomere (red) co-localization (white) at 0- or 6-h recovery. (**A**) Confocal images of untreated Wt cells, and Wt and XPV cells exposed to UVC or Cr(VI) and allowed to recover for 6 h. Average DDR foci and telomere foci per cell after 0 or 6 h recovery from UVC (**B**) or Cr(VI) (**C**). Data represent averages from two independent experiments and a minimum of 50 cells. Bars with a symbol of * indicates a significant difference compared to mock exposure and between the various recovery time points (*P* < 0.05). Bars, 10 μM. Untreated, UT.

Stalled replication forks at blocking DNA lesions can collapse into a DNA DSB, potentially due to cleavage of single stranded DNA at the stalled fork by endonuclease or spontaneous breakage (8). Previous studies show both UVC and Cr(VI) exposure induce chromosome breaks that depend on S-phase progression and genome replication (27,35,36,45–47). These studies show proteins that signal a DNA damage response (DDR) and DSBs, including phosphorylation of histone H2AX (γH2AX) and p53-binding protein 1 (53BP1), form foci after UVC and Cr(VI) exposures in a manner that requires S-phase progression. Since polη was shown to suppress γH2AX response after UVC exposures (45,46), we asked if polη also prevents DDR signaling at telomeres following the genotoxic exposures. Wt or XPV cells were exposed to 5 J/m^2^ UVC and then fixed either 0 or 6 h after recovery in fresh media. Six hours were selected based on evidence for S-phase checkpoint activation for both agents at this time point (Figure 2). Given that γH2AX can also form at non-DSB sites (45), we identified DDR-positive telomeres as foci containing triple co-localized γH2AX, 53BP1 and telomeric DNA using the IF-FISH assay and confocal microscopy. We used an unbiased approach of including both small 53BP1 foci and large 53BP1 bodies (48), but the majority were small foci (data not shown). Both exposures induced 53BP1 foci formation (Supplementary Figure S3). UVC did not induce a significant increase in DDR+ telomeres immediately following exposures for either cell line (Figure 5). However, we observed a 3.3-fold increase in DDR+ telomeres at 6-h recovery in Wt cells and a larger than 6.8-fold increase in XPV cells, compared to untreated cells (Figure 5). At the 6-h recovery time point, cells lacking polη harbored a 2-fold increase in DDR+ telomeres compared to Wt. Following 48-h exposure to 3 μM Cr(VI), Wt cells showed a 2.8- and 3.7-fold increase in DDR+ telomeres at zero and 6-h recovery, respectively, compared to untreated (Figure 5). Moreover, XPV cells showed a 4.5-fold and 6.5-fold increase in DDR+ telomeres at 0- and 6-h recovery, respectively, compared to untreated. The number of DDR+ telomeres was greater in XPV cells compared to Wt at both recovery time points. The difference in quantifiable sites of DNA damage at telomeres between cells proficient and deficient in polη provide further evidence for a fundamental role of TLS in protecting telomeres.

### Polη protects against UVC and Cr(VI) induced telomere aberrations

**Figure 6.**
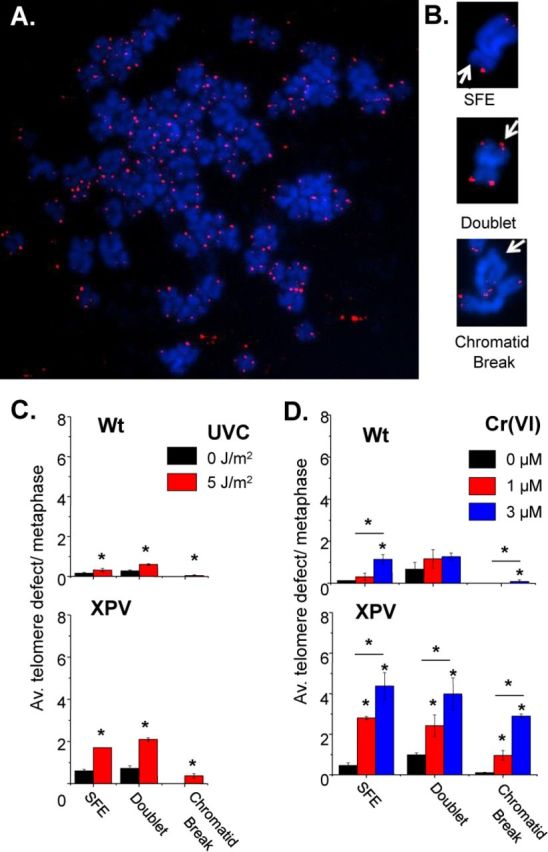
UVC and Cr(VI) induce telomere aberrations in XPV cells. (**A**) Representative metaphase of telomere FISH of untreated Wt cells. (**B**) Representative images of telomere aberrations and chromatid breaks. SFE, signal-free end. (**C**) Average telomere defect per metaphase after 0 or 5 J/m^2^ UVC irradiation, 6 h recovery and 8 h colcemid or (**D**) 0, 1 or 3 μM Cr(VI) for 48 and 8 h colcemid. Bars with * are significantly different (*P* < 0.05). The data represent mean ± SE from two individual experiments with approximately 50 metaphases. Telomere signal-free ends, SFE.

Having established that polη localizes to telomeres and suppresses DDR signaling at telomeres after UVC and Cr(VI) treatments, we next asked whether polη functions in preserving telomere structure and integrity following genotoxic exposure. We previously reported that Cr(VI)-induced replication stress leads to telomere aberrations in human fibroblasts (27). While exposing human fibroblasts to UVB failed to alter mean telomere lengths, the impact on individual telomeres had not been examined (24). To examine polη function in preserving telomeres structure after the exposures, we prepared and stained chromosome metaphase spreads for telomeres by fluorescent *in situ* hybridization (Telo-FISH) (Figure 6A-B). Following 6-h recovery from UVC exposure, cells were treated with colcemid for 8 h to arrest cells in metaphase. Since this time point coincides with active DNA synthesis in the cells that received 5 J/m^2^ UVC, but not 10 J/m^2^ (Figure 2), we reasoned that only the lower dose would allow the cells to reach metaphase within the experimental time frame. Interestingly, the mock treated XPV cells exhibited 3.7-fold more signal free ends (SFEs) and 2-fold more telomere doublets, compared to mock Wt cells (Figure 6C). This may be related to polη roles in bypass of oxidative damage and/or fragile site stability (13,22). UVC exposure of Wt cells induced a 2-fold increase in telomere aberrations, although averaging less than one aberration per metaphase for both telomere losses and doublets (Figure 6C). However, XPV cells showed a significant increase in telomere losses and doublets (about 3-fold each) after 5 J/m^2^ when compared to untreated cells. Additionally, we observed UVC induces chromatid breaks in polη-deficient cells consistent with previous reports (20) and confirming polη's important role in chromosome stability. Similar results were obtained in primary skin fibroblasts, BJ and XPV (GM02359), exposed to 0 and 5 J/m^2^ UVC (Supplementary Figure S4).

We examined telomeres following 1 and 3 μM Cr(VI), since exposures at these concentrations revealed cell cycle progression during the recovery period required to obtain metaphase cells for chromosomal analysis (Figure 2). Colcemid was applied immediately following the 48 h of Cr(VI) exposure. Similar to UVC, the mock treated XPV cells exhibited a higher level of telomere loss and telomere doublets, compared to Wt (Figure 6D). Cr(VI) exposures induced a concentration dependent increase in both telomeres losses and doublets for the Wt cells, although the total aberrations per metaphase remain close to one. Strikingly, we observed a greater Cr(VI) induction of telomere aberrations for the XPV cells compared to Wt, in most cases. The 1 μM Cr(VI) exposure of XPV cells induced a 6-fold increase in telomere losses and a 2.5-fold increase in doublets compared to mock. The 3 μM Cr(VI) caused a 9.5-fold increase in telomere losses and a 4-fold increase in doublets compared to mock. Notably, these two types of aberrations are associated with replication stress, while our findings of telomere chromosome or chromatid fusions were less than 0.05 and 0.25 in 50 metaphases analyzed for 5 J/m^2^ UVC and 3 μM Cr(VI), respectively, in XPV cells (data not shown). Similar to UVC, we also observed that polη suppressed Cr(VI)-induced chromatid breaks illustrating a role for polη at non-telomeric regions following Cr(VI) exposures as well (Figure 6D).

Our findings are a first account of a role for polη in preserving telomere integrity after relatively low levels of UVC or Cr(VI) exposure. The dramatic increase in replication-associated telomere aberrations in cells lacking functional polη compared to Wt cells suggests that polη is required for proper replication of telomeres following the induction of bulky DNA adducts.

## DISCUSSION

We uncovered a novel role for polη in telomere preservation. Previous studies have shown that various DNA repair pathways are either reduced or suppressed at telomeres (1,9,24). Our findings provide strong evidence that TLS polη gains access to, and functions at telomeres, after the induction of bulky DNA lesions. TLS represents a DNA damage tolerance pathway that does not repair the damage, but defends the genome against consequences of unrepaired DNA lesions. Polη's role in TLS prevents stalled replication forks from collapsing into DSBs through its ability to bypass DNA lesions during replication. Our data provide new evidence that telomeres rely on lesion bypass mechanisms for replication after genotoxic stress, consistent with reports that alternative mechanisms of fork recovery including HR and DSB repair are normally suppressed at telomeres (1,9).

### Polη roles after Cr(VI) exposure

Polη's role in lesion bypass extends beyond UV-induced CPDs to roles in normal replication after hydroxyurea (32), cisplatin and gemcitabine (33). O'Brien *et al.* showed that polη-mediated TLS prevents Cr(VI) induced mutations in *S. cerevisiae* (28). Here, we report the first evidence that polη protects against Cr(VI) exposure in mammalian cells. Polη-deficient human cells exhibited hypersensitivity and increased chromatid breaks (Figures 1 and 6). Furthermore, Cr(VI) induced polη foci formation similar to UVC (Figure 4). These data suggest that polη functions to bypass Cr(VI) lesions during replication in a similar fashion as CPDs. UV photoproducts are bulky lesions that distort the double helix and stall replication forks (15). Cr(VI) forms a spectrum of DNA lesions, most of which are bulky binary or ternary Cr-DNA adducts bound to the phosphodiester DNA backbone (49), which impede polymerase progression (34). Nucleotide excision repair (NER) removes photoproducts (50) and Cr-DNA adducts (25). Therefore, it is not surprising that TLS mechanisms operate at Cr-DNA adducts, similar to UV photoproducts to prevent replication fork collapse at unrepaired lesions.

Cell cycle analysis revealed that polη functions in normal recovery from Cr(VI)-induced replication inhibition (Figure 2). Control experiments with UVC confirmed that cells lacking polη were delayed in S-phase, based on fewer cells synthesizing DNA during recovery compared to Wt cells (Figure 2) (18,29). The pattern of EdU-positive cells differs for Cr(VI) compared to UVC (Figure 2B), and we attribute this to an acute physical versus chronic chemical exposure for UVC and Cr(VI), respectively. At 0 h recovery XPV cells exposed to 3 μM Cr(VI) show reduced EdU-positive cells, whereas reductions were not observed until 6 h recovery from UVC. S-phase checkpoint activation likely occurred during the 48-h Cr(VI) exposure, but would require time for replication forks to encounter UV lesions after the acute irradiation. We interpret the reduction in EdU-positive XPV cells after Cr(VI) as a Cr(VI)-induced S-phase delay, similar to UVC, because significant cell death did not occur during recovery (Supplementary Figure S1). Consistent with this, we observe cell proliferation in both Wt and XPV cells 8 days post exposure (Figure 1). However, the lack of full recovery by 24 h following 3 μM Cr(VI) exposures of XPV cells suggests a fraction of these cells remain arrested.

### Evidence that bulky lesions induce fork stalling at telomeres

UV irradiation and Cr(VI) exposure revealed concentration-dependent increases in ATR foci and polη per cell (Figure 3). We propose these foci identify sites of replication stress at DNA lesions and contribute to signaling the S-phase checkpoint based on previous reports. Blocked replication forks produce ssDNA intermediates provoking RPA-mediated ATR recruitment during S-phase (40,43), and ATR mediates S-phase checkpoint signaling in response to ssDNA intermediates (40). S-phase checkpoint activation inhibits DNA synthesis as cell cycle progression pauses to repair the damage, which is consistent with the cell cycle profiles following UVC and Cr(VI) exposure (Figure 2). The induction of both ATR and polη foci at telomeres in response to UVC and Cr(VI) exposures (Figures 3 and 4) suggests that replication forks are stalled at telomeres due to unrepaired lesions blocking the forks. Previous studies show polη translocates to stalled replication forks and polη foci overlap with CPD antibody staining (44). Furthermore, CPDs were detected at telomeres following UVC exposures (23,24) (our unpublished data). Although the foci counts are low per cell for both ATR and polη at telomeres, we propose they are significant. Confocal microscopy displays one plane of focus of the nucleus where on average 20 telomere foci are visible in our images. Of these foci, about 14% co-localized with polη foci after 10 J/m^2^ UVC and 9% co-localized with polη foci after 3 μM Cr(VI) (Figure 4). Conversely, about 12 or 14% of polη foci localized to telomeres after 10 J/m^2^ UVC or 3 μM Cr(VI), respectively. Given that telomeres comprise less than 0.025% of the genome, we propose this represents a striking TLS response.

### Polη suppression of DDR at telomeres after bulky lesion production

Signaling of the DNA damage response (DDR) at telomeres may signify DSB formation and/or unprotected and dysfunctional telomeres. The DDR is normally suppressed at functioning telomeres, but is activated when telomeres are deprotected upon loss of structure or the shelterin complex (2). Unprotected telomeres are vulnerable to inappropriate DNA repair and chromosome fusions because they are physically similar to DSBs (1). DDR also occurs when stalled forks collapse into DSBs (45,46), which may cause telomere loss based on reports that DSBs are not repaired at telomeres (9). XPV cells show more cells with γH2AX foci after UV, hydroxyurea and psoralens, and increased activation of ATR after UV (32,45,51,52). In agreement with these reports we found polη suppresses global DDR and decreased DDR at telomeres after UVC and Cr(VI) (Figure 5). We propose the telomeric DDR arise from replication fork demise at telomeres because we and others showed γH2AX foci formation following UVC and Cr(VI) depend on S-phase progression (27,35,36,46,47). In contrast, DDR at telomeres due to shelterin loss does not depend on cell cycle (53), and the UVC and Cr(VI) lesions frequency is unlikely to be high enough to displace significant shelterin.

Consistent with previous reports (48,54), we observed two types of 53BP1 formations; small foci or large bodies. Small foci are typically more abundant than the large bodies and were found to occur during S-phase (54). While our analyses included both variations of 53BP1 foci, we observed the vast majority were smaller foci rather than larger bodies. Both types of formations indiscriminately co-localized to γH2AX (data not shown). Moreover, the pattern of small 53BP1 foci we observed after UVC and Cr(VI) resembled those formed after aphidicolin treatment or loss of shelterin protein, which causes foci formation and fork stalling at telomeres (3).

### Polη suppression of telomere aberrations caused by bulky lesion production

Telomere losses and telomere doublets have been reported as consequences of replication fork stalling at telomeres (3,4). Telomere losses, or critically short telomeres, are proposed to arise from telomeric breaks that occur in response to collapsed forks (7). Doublets are also termed fragile telomeres because they arise upon cellular treatments that induce breaks at common fragile site sequences (3,5). The molecular nature of telomere doublets remains unknown, but they are proposed to represent aberrantly condense chromatin due to regions of unreplicated ssDNA (3). We found the generation of replication blocking lesions also causes both forms of telomere aberrations and is significantly enhanced in cells lacking polη (Figure 6). This suggests that lesion bypass by polη resolves replication blocks at telomeres, thereby suppressing breaks and accumulation of ssDNA or aberrant replication intermediates. Notably, the stabilization of blocking G-quadruplex structures at telomeres also induces both telomere loss and doublets (6). We see an average of two telomere losses and doublets per metaphase after UVC and 2–5 telomeres losses and doublets after Cr(VI) (Figure 6). Several factors influence detections of telomere aberrations. (i) Measuring telomere aberrations on metaphase chromosomes requires cell cycle progression. Therefore, the aberrations in XPV cells may be underestimated due to the increased S-phase delay in polη-deficient cells after genotoxic exposures (Figure 2). (ii) Both unrepaired replication forks and dysfunctional telomeres can activate p53-mediated G2 checkpoints and prevent progression to mitosis (20,55). Previous reports indicate that SV40-transformed XPV fibroblasts are more sensitive to UV and show more UV-induced sister chromatid breaks compared to primary cells due to large T-antigen suppression of p53 (20). Consistent with this, we observed fewer UV-induced chromatid breaks in primary cells, and fewer UV-induced telomere loss in the primary BJ cells (Supplementary Figure S4). Since detection of telomere loss and doublets occurs when checkpoints fail to prevent cell progression to metaphase, they may be more apparent in p53-defective cells (56).

One possibility is that telomere aberrations result from global DNA synthesis inhibition due to signaling from stalled forks elsewhere in the genome, rather than due to stalled forks at telomeric DNA lesions. We do not favor this model for several reasons. First, low level chronic Cr(VI) exposures caused a modest decrease in cells replicating DNA compared to UVC (Figure 2), yet Cr(VI) induces more telomere aberrations and DDR-positive telomeres (Figure 6). Second, we and others have demonstrated that UV photoproducts form at telomeres following UV irradiation (unpublished data) (23,24). Third, previous studies reported that UV irradiation with a porous filter resulted in ATR and polη staining only at sites of UV-induced lesions (44,57,58), suggesting that UVC irradiation does not induce replication stress at sites lacking DNA lesions. Finally, if the telomere aberrations are caused by global DNA replication inhibition and not lesions at the telomeres, then we would expect the level of UVC and Cr(VI)-induced aberrant telomeres to be higher and more similar to aphidicolin treatment after which every telomere is affected (3). If individual lesions are causing the replication stress that leads to telomere defects, then only those telomeres with a lesion should be affected, and we would not expect every telomere would harbor blocking lesions. Lesion generation is random and stochastic. Aphidicolin affects all replication forks because the DNA polymerase is inhibited. Importantly, some telomeres with a lesion might be bypassed by other TLS polymerases, such as polymerase ι or polymerase ζ (13).

### Roles for polη in preserving telomeres in the absence of exogenous damage

The telomere aberration analysis also revealed that untreated XPV cells show an increase in telomere losses and doublets compared to untreated Wt cells (Figure 6). The difference for losses and doublets is significant for both the mock untreated samples in both the UVC and Cr(VI) experiments (Student's *t*-test, *P* < 0.05). Other studies have demonstrated that telomere doublets and aberrations result from endogenous damage. Both telomere losses and doublets were reported in cells lacking glycosylases that remove 8-oxo-guanine and oxidized pyrimidines (59,60) and that harbor unresolved G-quadruplexes (6). Previous studies report polη bypasses 8-oxoguanine and thymine glycol lesions (33) demonstrating the importance of polη in cells experiencing endogenous damage. Furthermore, polη-deficient cells are hypersensitive to ligands that stabilize G-quadruplex structures that can form in telomeric DNA (61). Combined with these previous studies, our work suggests that polη has a role at telomeres even at sites of endogenous lesions emphasizing polη as a requirement for telomere maintenance.

### Biological implications

Our study reveals the novel finding that polη protects against telomere defects after both an acute physical (UVC) and chronic chemical (Cr(VI)) exposure, and this role likely extends to the induction of bulky lesions from other sources capable of causing replication stress (33). Based on reports that telomeres lack robust DNA repair mechanisms compared to the rest of the genome (1,9,23,24), our data supports the model that telomeres, in particular, may rely heavily on TLS to avoid the consequences of replication fork collapse (i.e. DSB formation). Our data also uncover new evidence that UVC irradiation can induce telomere loss and fragility. This is significant in light of new studies that classify UV irradiation as an environmental geratogen based on evidence that UVB exposure induces cell senescence in irradiated *p16*-reporter mice (62). Our findings provide evidence that UV light and the consequent DNA photoproducts may promote senescence and aging in part by disrupting telomeres that harbor the lesions.

## SUPPLEMENTARY DATA

Supplementary Data are available at NAR Online.

SUPPLEMENTARY DATA
