# Supplementary material for: Polymerase η suppresses telomere defects induced by DNA damaging agents
Source: Nucleic Acids Res. 2014 Oct 29;42(21):13096–109. doi: 10.1093/nar/gku1030 (PMC4245935; doi:10.1093/nar/gku1030)
Supplement: SUPPLEMENTARY DATA [file supp_42_21_13096__index.html]

Polymerase η suppresses telomere defects induced by DNA damaging agents — SUPPLEMENTARY DATA 

# Polymerase η suppresses telomere defects induced by DNA damaging agents

## SUPPLEMENTARY DATA

**Files in this Data Supplement:**

- SUPPLEMENTARY DATA
