# Supplementary material for: Polymerase η suppresses telomere defects induced by DNA damaging agents
Source: Nucleic Acids Res. 2014 Oct 29;42(21):13096–109. doi: 10.1093/nar/gku1030 (PMC4245935; doi:10.1093/nar/gku1030)
Supplement: SUPPLEMENTARY DATA [file supp_gku1030_nar-02617-d-2014-File008.pdf]

Supplemental Figure Legends

Figure S1. Viability test of Wt and XPV cells after UVC exposure or Cr(VI) exposure. Cell viability was evaluated of SV40 immortalized XP30RO-derived cells expressing polη (Wt) or vector alone (XPV) prior to cell cycle profiling with LIVE/DEAD® Fixable Dead Cell Staining Kit. Cells were (A) irradiated with 0, 5, or 10 J/m^2 UVC or (B) exposed to 0, 1, or 3 µM Cr(VI) for 48 h and then recovery in fresh media for 48 h.

Figure S2. UVC induces polymerase η localization to telomeres in U2OS cells. (A) Confocal images of EGFP-Polη U2OS cells exposed to UVC and recovered for 6 h. Cells were analyzed via IF-FISH of polη (green) and telomere (red) co-localization (yellow). (B) Average polη foci and co-localized polη and telomere foci per cell after indicated UVC dose. The data represent mean ± SE from two individual experiments and a minimum of 100 interphase cells. Bars with a symbol of * indicates a significant difference compared to mock exposure and between the different concentrations, black bars refer to Polη foci, red bars refer to Polη + Telomeres ($p<0.05$). Bars, 10 µM.

Figure S3. UVC and Cr(VI) induce 53BP1 foci formation. SV40 immortalized XP30RO-derived cells expressing polη (Wt) or vector alone (XPV) following irradiation with 5 J/m^2 UVC and recovered for six hours or exposure to 3 µM Cr(VI) for 48 h. Cells were analyzed via IF-FISH for 53BP1 at 0 or 6 hours recovery for average 53BP1 foci per cell after UVC (A) or Cr(VI) (B). Data represent averages from two individual experiments and a minimum of 50 cells. Bars with a symbol of * indicates a significant difference compared to mock exposure and between the two concentrations ($p<0.05$). Bars, 10 µM. Untreated, UT.

Figure S4. UVC induces telomere aberrations in BJ and GM02359 cells. (A) BJ and (B) XPV (GM02359) primary fibroblasts exposed to 0 or 5 J/m^2 UVC, recovered for 6 h, and incubated for 10 h with colcemid. Analysis of telomere defects per metaphase was evaluated using telomere FISH. Bars with * are significantly different ($p<0.05$). The data represent mean ± SE from an average of two to three independent experiments and 75 metaphases. Telomere signal free ends, SFE.

Figure S1

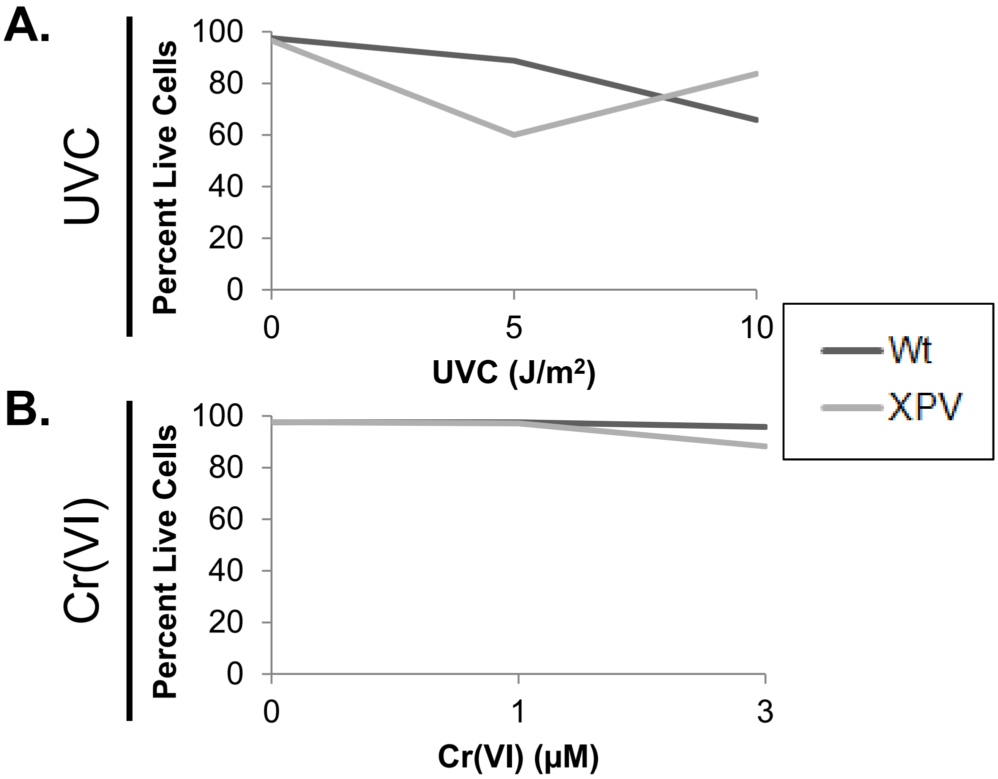
A.
UVC
Percent Live Cells
100
80
60
40
20
0
0
5
10
UVC (J/m²)
Wt
XPV
B.
Cr(VI)
Percent Live Cells
100
80
60
40
20
0
0
1
3
Cr(VI) (µM)

Figure S2

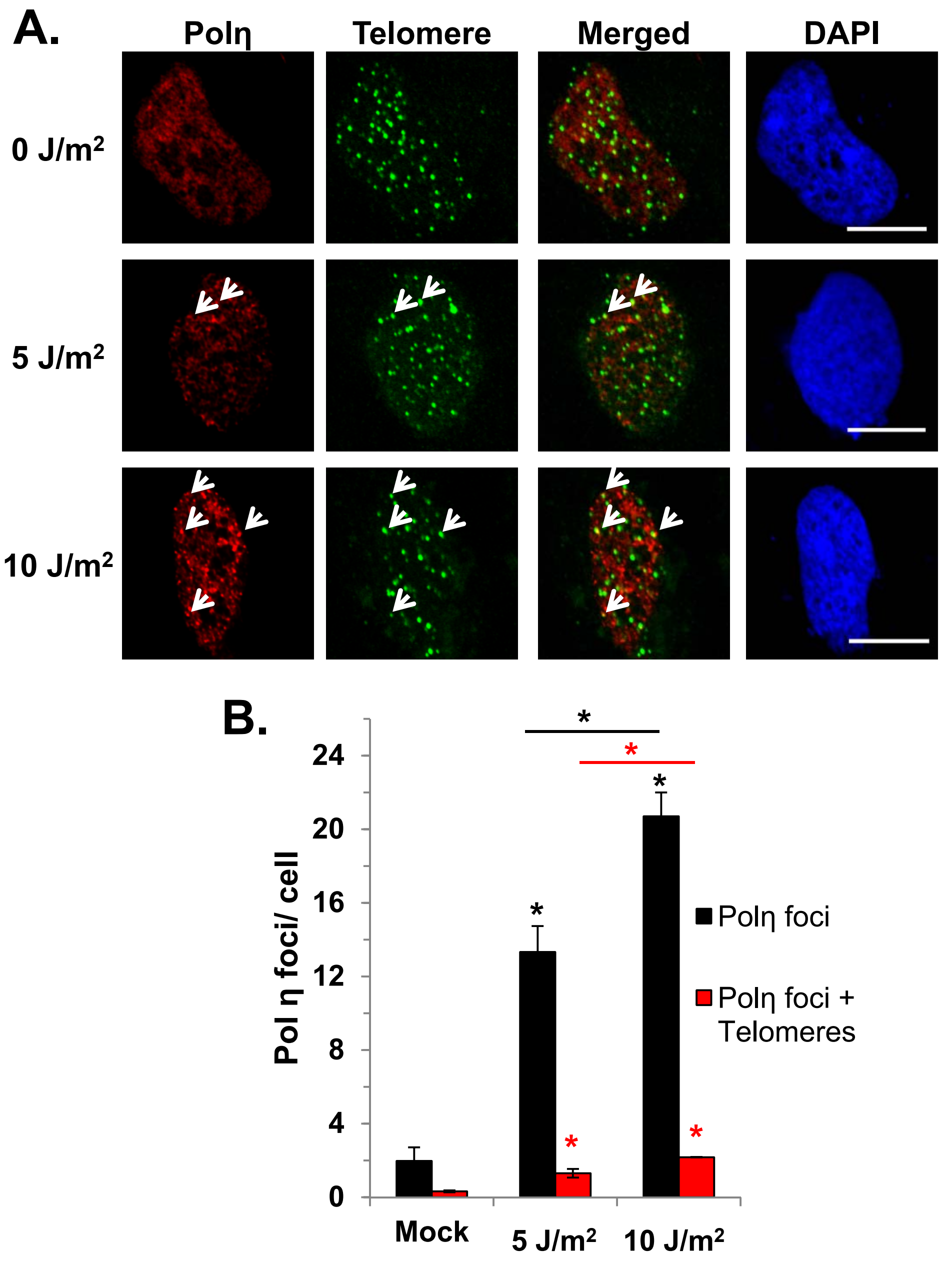

A.
Polη
Telomere
Merged
DAPI
0 J/m2
5 J/m2
10 J/m2
B.
Pol η foci/ cell
24
20
16
12
8
4
0
Polη foci
Polη foci + Telomeres
Mock
5 J/m2
10 J/m2

Figure S3

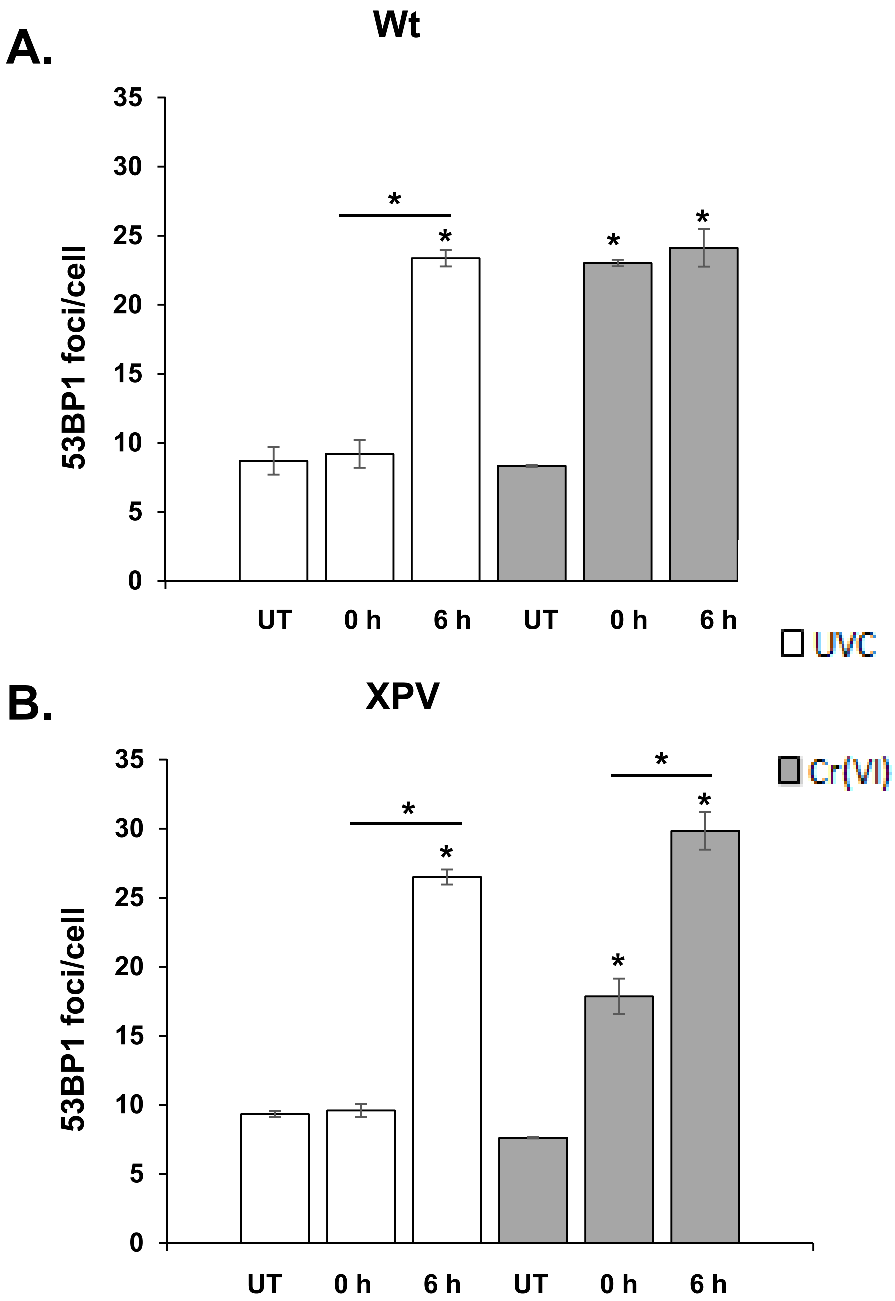
A.
Wt
53BP1 foci/cell
35
30
25
20
15
10
5
0
*
UT
0 h
6 h
UT
0 h
6 h
UVC
B.
XPV
Cr(VI)

Figure S4

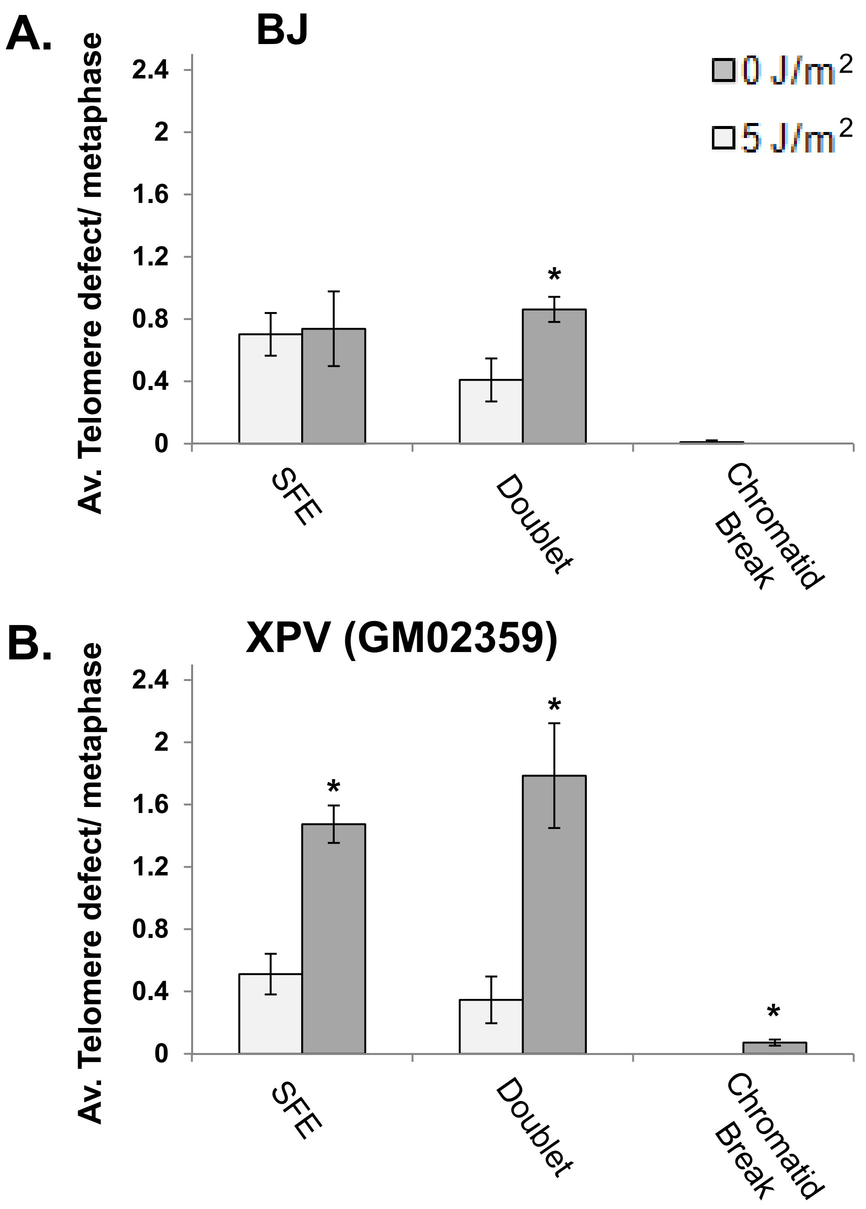
A.
BJ
0 J/m2
5 J/m2
Av. Telomere defect/ metaphase
2.4
2
1.6
1.2
0.8
0.4
0
*
SFE
Doublet
Chromatid Break
B.
XPV (GM02359)
Av. Telomere defect/ metaphase
2.4
2
1.6
1.2
0.8
0.4
0
*
*
*
SFE
Doublet
Chromatid Break
